# TomatoweedDet: a real-field multi-class weed detection dataset and YOLO benchmark for tomato production systems

**DOI:** 10.3389/fpls.2026.1861020

**Published:** 2026-06-18

**Authors:** Alperen Mehmet Sevinç, Çağrı Vakkas Yıldırım, Ahmet Kerim Ağırman

**Affiliations:** 1 Department of Aircraft Technology, School of Ankara Aeronautical Vocational Higher Education, University of Turkish Aeronautical Association, Ankara, Türkiye; 2 Erciyes University, Graduate School of Natural and Applied Sciences, Aviation Electrics and Electronics, Kayseri, Türkiye; 3 Department of Aircraft Airframe And Powerplant Maintenance, Faculty of Aeronautics and Astronautics, Erciyes University, Kayseri, Türkiye; 4 Department of Aviation Electrics and Electronics, Faculty of Aeronautics and Astronautics, Erciyes University, Kayseri, Türkiye

**Keywords:** deep learning, machine learning, precision agriculture, site-specific weed management, smart agriculture, weed detection

## Abstract

This study presents an approach for the object detection of multiple weeds in tomato production systems based on deep learning. A comprehensive dataset has been collected in three provinces of Türkiye (Balıkesir, Ankara, and Aksaray) under real-world field conditions. The data set has 32,607 images and 44,165 bounding boxes annotations. The two weed species included in the dataset are, to our knowledge, underrepresented in the current deep learning-based agricultural object detection literature. Drone and smartphone cameras took pictures at different times of the day (morning, noon, and afternoon) of different soil textures, light levels, and weather conditions, such as rain, mud, and shadows. The dataset reflects agricultural diversity as it exists in the real world, unlike previous studies that relied on controlled experimental environments. The model was trained using YOLO-based deep learning algorithms within the PyTorch framework. The metrics Precision, Recall, mAP@0.5, and mAP@[0.5:0.95] were used to evaluate the performance of the models. In this study, seven different YOLO architectures were comparatively evaluated on the TomatoWeedDet dataset created under real field conditions. The results show that the YOLOv8l model demonstrates high performance in the multi-class weed detection task and has significant potential for precision weed management applications. The model that was created could be used in mobile or embedded systems to monitor weeds in real time with drones. The proposed system enables targeted herbicide application and less use of chemicals. This study advances research on weed detection using deep learning. It also helps to make precision and sustainable farming systems a reality.

## Introduction

Tomatoes (*Solanum lycopersicum L*.) are one of the world’s most important vegetable crops due to their widespread cultivation and high profitability. It has a significant position in the agricultural economy since it is extensively utilized in the food business, there is significant domestic demand for it, and it has strong export potential. In 2023, Türkiye was the third greatest producer of tomatoes in the world, after China and India. FAO (2024) said that the world has grown more than 190 million tons of tomatoes. Türkiye grows over 4.5 million tons of tomatoes every year. This is about 40% of all the vegetables farmed in the country. Türkiye’s agricultural exports bring in a lot of money, and tomatoes are a big part of that. Türkiye earned over US$750 million in revenue in 2023 (Ministry of Trade, 2024). As a result, it is important to enhance tomato quality for the national economy and future of agriculture. Türkiye produces 4.6 million tons of apples and 5.2 million tons of potatoes. This highlights the importance of tomatoes in Turkish agriculture (Turkish Statistical Institute (TÜİK), 2024).

The most important element that reduces the tomato yield is weeds. Weeds occupy the space, water, nutrients, light, other resources that plants need, slowing their growth and negatively impacting yield (Khaffagy et al., 2022). Weeds are also a reservoir of many plant pathogens and hosts, thereby facilitating the spread of plant diseases in the field (Brandsæter et al., 2020). These damages are lasting and, above all, they intervene during the phase of growth of the tomato unit. There are seven common weeds in tomato fields: *Acroptilon repens (L.)* (acı ot), *Amaranthus retroflexus L.* (horoz ibiği), *Chenopodium album L.* (sirken otu), *Fallopia convolvulus* (tarla sarmaşığı), *Fumaria parviflora* (tarla şahteresi), *Orobanche* spp. (canavar otu), and *Sinapis arvensis L.* (hardal otu). Weeds rapidly grow and spread, being competitive during the vegetative phase.

*Acroptilon repens (L.)* Theiler is characterized by its wide-sweeping root system and allelopathic compounds. It suppresses plant growth and changes soil microorganisms as well as inhibits the development of plant roots (Foxx et al., 2024; Hassan et al., 2024; Regensberg, 2007). *Amaranthus retroflexus L.*, known for its rapid adaptation ability, can reduce yield by up to 35% in both garden and greenhouse cultivation (Margaryan et al., 2025). *Chenopodium album L.* negatively inhibits seedling development in immature tomato plants by reducing photosynthetic efficiency (Zaragoza et al., 2003). If weeds are not removed, nearly 50% of the tomato crop can be lost (Prakash and Srivastva, 2006). *Fallopia convolvulus* and *Fumaria parviflora* form a thick soil cover, preventing plants from absorbing water and nutrients. Root secretions also weaken the soil biota and inhibit seedling growth (Tei et al., 2003). *Orobanche* spp. is a semi-parasitic weed that uses the roots of host plants as a food source. It is predicted to be the most harmful weed for tomato cultivation (Parker, 2009). *Sinapis arvensis L.*, encountered in many crops, grows and reproduces rapidly despite chemical control. This is because it has a large number of seeds (Warwick et al., 2000).

Using herbicides is still the most common way to get rid of weeds. However, unnecessary and incorrect herbicide use harms soil microorganisms, pollutes water sources, and reduces the effectiveness of herbicides (Parven et al., 2025). Herbicides are much less effective if used after the weeds have grown. Early detection of weeds protects nature and makes weed control more effective (Nedeljković et al., 2025). As weeds grow, they develop stronger roots, which reduces the effectiveness of mechanical control methods.

**Figure 1:**
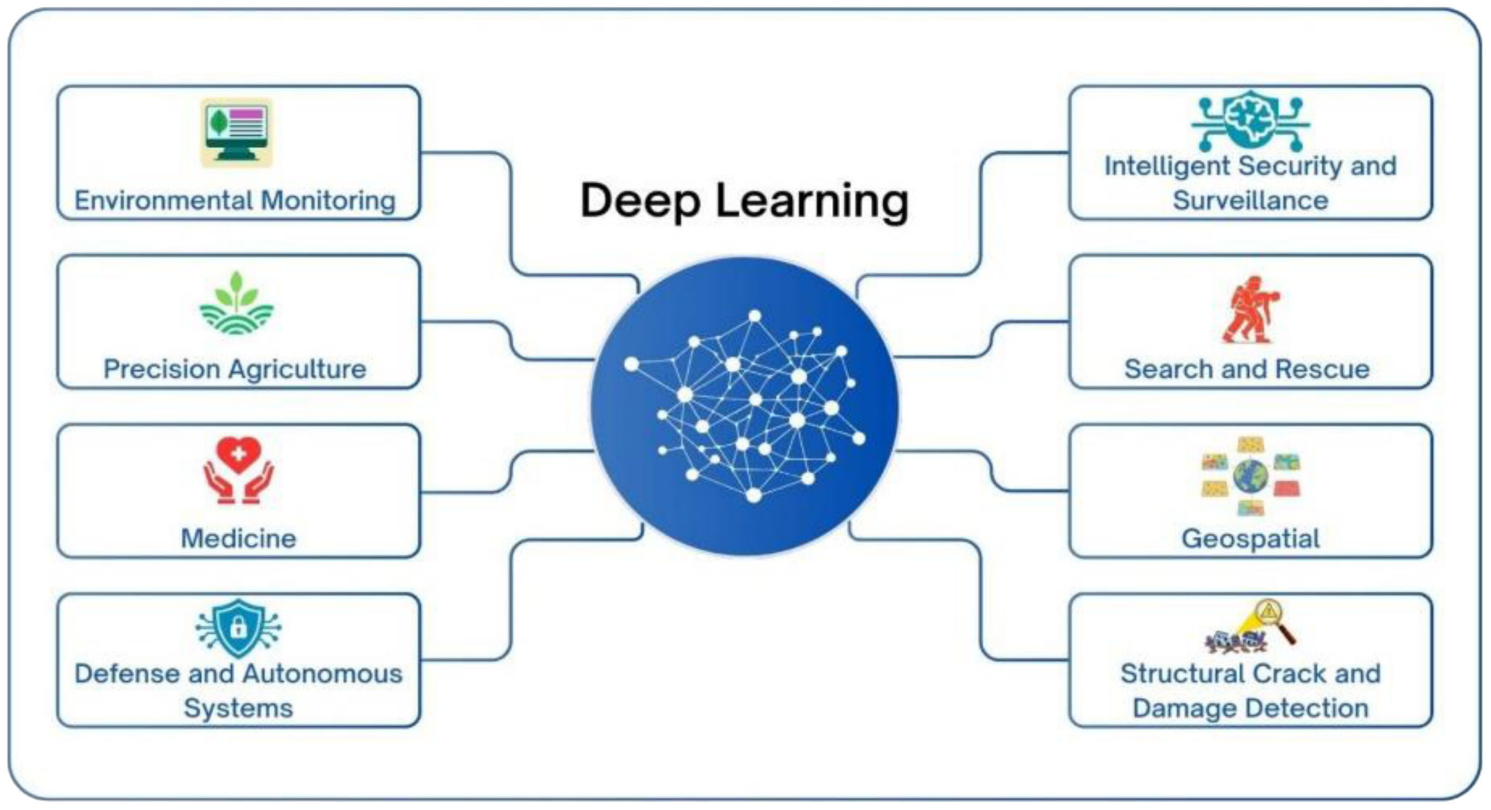
Places where deep learning is often used.

In the last few years, methods based on image processing and deep learning have shown a lot of promise for finding weeds early on. Deep learning is a way to learn that uses multilayered neural networks to copy how the human brain learns (Aggarwal, 2023). The framework of deep learning is being increasingly applied to various scientific and technological fields due to its flexible and useful structure. Such applications include medicine (Esteva et al., 2017; Litjens et al., 2017), geospatial and remote sensing (Coulson et al., 2025; Zhu et al., 2017), environmental monitoring (Reichstein et al., 2019), search and rescue operations (Ciccone and Ceruti, 2025), structural crack and damage detection (Cha et al., 2017; Dorafshan et al., 2018), defense and autonomous systems (Kim and Joe, 2025)as well as intelligent security/surveillance (Abid et al., 2024) and precision farming (Kamilaris and Prenafeta-Boldú, 2018). This technique has been successfully employed in numerous agricultural tasks, such as plant classification, disease detecting, yield forecasting, fruit ripeness evaluation and weed recognition (Darwin et al., 2021). **Figure 1** shows these areas. It’s not surprising that Convolutional Neural Networks (CNNs) are well-suited to getting things right, and the reason is clear they can retrieve spatial features straight from visual data. Since deep architectures are quite heavy, it is very important to design efficient and lightweight models, which can be used in real time.

The YOLO (You Only Look Once) family of object detectors is now widely used. Redmon et al. (2016) primarily proposed YOLO models. Such models have demonstrated high precision and the capability of real time under a single stage detection pipeline, that conducts both classification and localization at the same time. Recent flavors like YOLOv3, YOLOv5, YOLOv7, YOLOv8 and YOLOv10 are increasingly popular in agricultural imaging (Jocher et al., 2020; Redmon et al., 2016; Wang et al., 2022). Compared with all other methods, YOLOv8 achieves the best performance gains related to optimized backbone, refined anchor free mechanism and more efficient feature merging layers. This is particularly interesting for the detection of small objects and for multiple weed types detection, making algorithm a very successful method (Dang et al., 2023).

YOLOv8 comes with multiple sub-models (n, s, m, l, x) that trade off between model size and the computational time. This potential feature renders it beneficial for farms with little equipment. In this research, seven YOLO variants (YOLOv3-Tiny, YOLOv5s, YOLOv8n, YOLOv8s, YOLOv8m, YOLOv8l, and YOLOv10n) were evaluated in terms of their capacity to detect and differentiate eight plant species (seven weed species and tomatoes) in tomato fields. Notably, among all the models tested, YOLOv8l model was observed to perform best in terms of P, R, and mAP, and was generally a superior model. These results confirm previous studies; for example, Dang et al. (2023) similarly stated that YOLOv8 showed improved efficacy in detecting small objects in cotton fields. Consequently, the YOLOv8l model represents the most suitable configuration for TomatoWeedDet dataset’s detection in tomato cultivation, combining high accuracy and practical usability.

Looking at the current literature, it is seen that many studies have been conducted on different types of crops and weeds. However, despite their detrimental effects on crops, some weed species have yet to be studied for deep learning-based detection. This study provides resources for a multi-environmental field study and a large-scale tomato weed control study, adapted to real field conditions. Seven weed species frequently found in tomato fields are particularly dangerous, as they not only reduce yield but also damage soil health. Yet, a literature review shows that most studies on deep learning or image processing focus on species such as *Amaranthus retroflexus L., Chenopodium album L., Orobanche* spp.*, Fallopia convolvulus* and *Sinapis arvensis L.* To the best of our knowledge, *Acroptilon repens (L.)* and *Fumaria parviflora* have received limited attention in previous deep learning-based weed detection studies. This highlights a significant gap in research on deep learning-based weed recognition systems. This study not only focuses on identifying the seven most common weed species that grow alongside tomatoes but also represents large-scale application designed to visually identify two previously unstudied (image and deep learning-based approaches) weed species. One of the key contributions of this study is the presentation of a large-scale, multi-class weed dataset generated under real field conditions. The dataset is designed to include different soil structures, environmental conditions, viewing angles, and natural agricultural variations. In addition, the study provides a comparative reference infrastructure for future deep learning-based weed detection systems by enabling the systematic evaluation of different YOLO architectures under the same experimental conditions. The following sections provide a detailed description of the materials, methods, and systems used, as well as the results obtained.

## Materials and methods

### Field study and data collection

**Figure 2:**
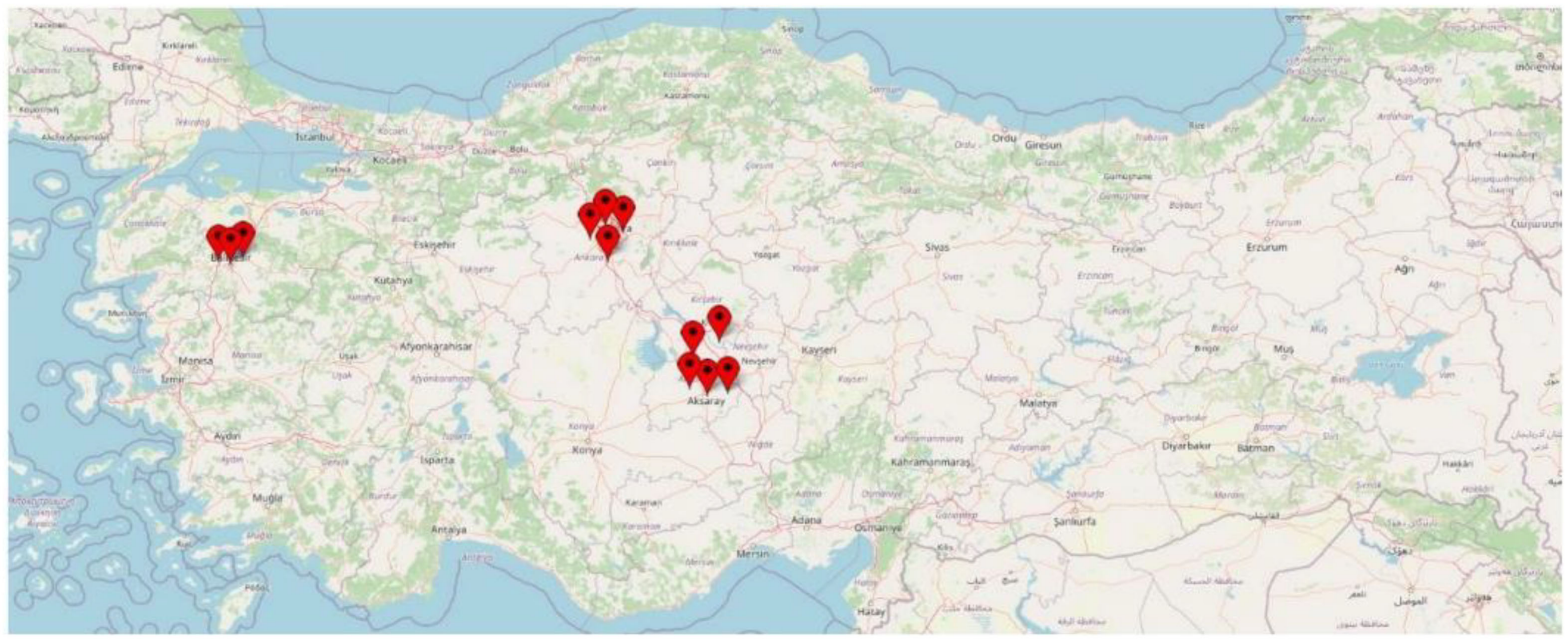
Map of fields where weeds and tomato plants were obtained.

This field study was conducted in three provinces of Türkiye to collect a weed dataset reflecting real field conditions and to support precision farming. These three provinces are Balıkesir (Kepsut Hotaşlar and Nusret), Ankara (Batıkent, Başayaş, Gölbaşı Hacılar and Bala Afşar), and Aksaray (Çatalsu, Kurtuluş, Lalebağı, Kederli and Armutlu) villages. **Figure 2** shows all the fields where the photographs were taken and a detailed map of the study areas. While collecting the data, efforts were made to create environments that were as similar as possible to real field conditions. No controlled testing environment was created. The entire dataset was collected from real agricultural environments showing various soil types, light conditions, damaged plants, and irrigated/dry soil conditions common in open-field tomato cultivation in Türkiye. The collected data has been gathered in a heterogeneous manner, and this method represents real field conditions, enabling the dataset to provide more realistic results.

To obtain realistic ground-level data, an iPhone 11, iPhone 14 Pro Max, and a professional-grade DJI Mavic 3 Pro drone camera were used. We obtained 32,607 images. This gave us a total of 44,165 bounding box annotations. 15,207 images (46.42%) were obtained with the drone.

To make the model more realistic and ensure the dataset yielded realistic results, the dataset was collected under different lighting and environmental conditions, such as morning, midday, and afternoon light. Weed data were captured at all growth stages, from small seedlings to adult seedlings. The dataset also included samples that were weathered, rotten, hail damaged muddy, withered, and taken from different angles (e.g., top-down, side, and oblique views). The intention is to make the dataset less homogeneous, and therefore more realistic and easier for the model to generalize to real agriculture settings. These realistic approaches could deteriorate the performance of the model on precision, recall and mAP. This was a deliberate decision to obviate the use of controlled experiment accuracy and reflect practical field performance (Maxwell et al., 2021).

The data, belonging to each class of tomato or weed, was bounding boxes annotations using IrfanView64 software. Species identifications and bounding box annotations were created while verifying agricultural weed species. The images were re-checked, particularly to minimize mislabeling of morphologically similar species. 44,165 bounding boxes annotations objects were identified in 32,607 images.

To prevent potential confusion due to long scientific names during training, each plant species was assigned a unique number. These numbers are 0, 1, 2, 3, 4, 5, 6, 7 respectively, and they have made the training process more organized.

**Figure 3:**
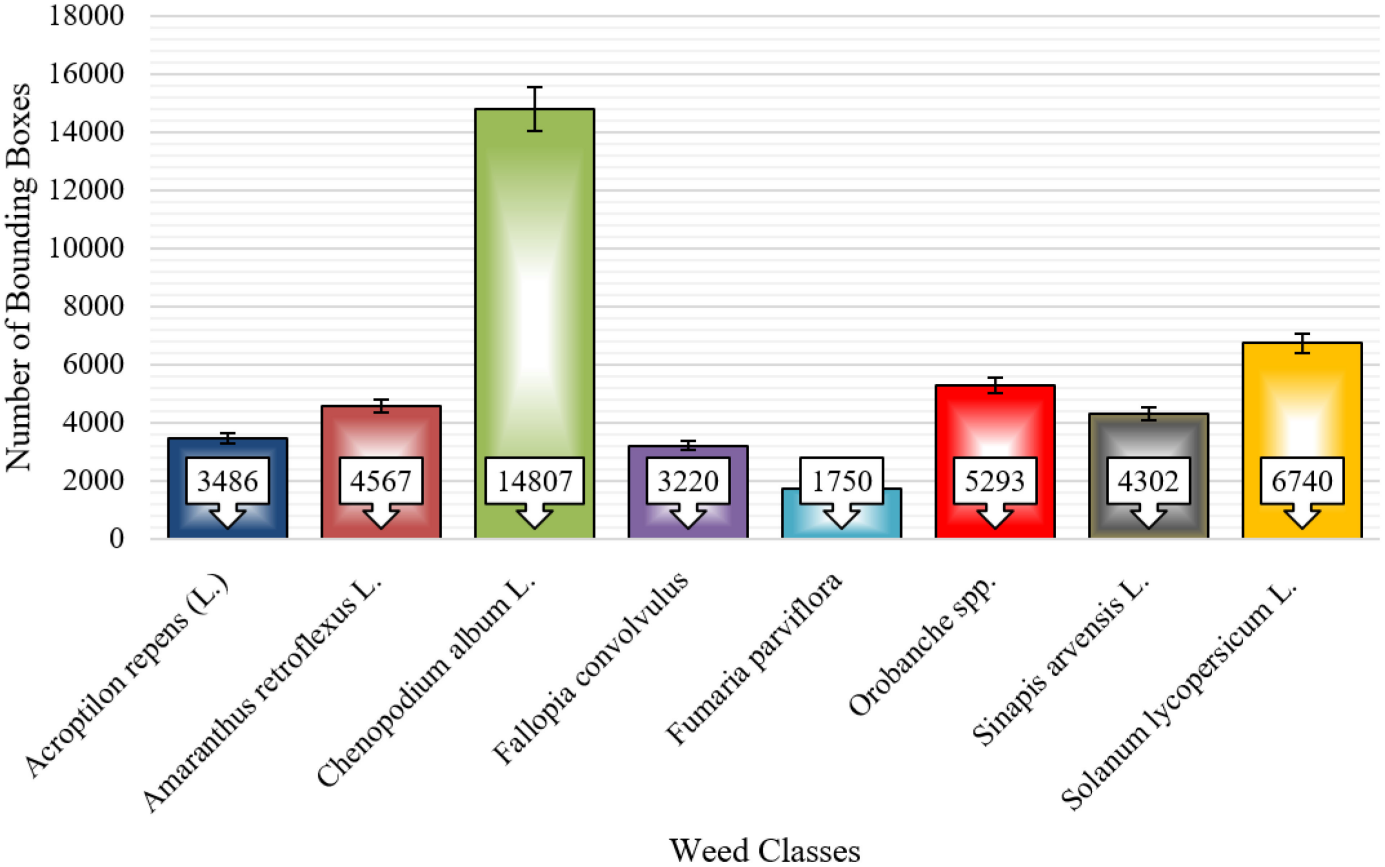
Bar graph of data for 8 tomato-weed classes, consisting of 32,607 images and 44,165 bounding boxes.

The classes were defined as follows:

**Table 1** shows that 44,165 labels were created on 32,607 images. The labeling process is based on the scientific classification of plant species. Weed species with similar characteristics in the field were placed in the same class. This method was designed so that the model would not have problems with visual differences in different field conditions and would generalize well at the class level. **Figure 3** shows the number of visual and limiting box labels for weed species.

**Table 1:** Alphabetical codes assigned to weed species, along with the number of labels and images for each class.

Class	Scientific name	Number of label	Number of images
0	*Acroptilon repens* (L.)	3.486	2.593
1	*Amaranthus retroflexus* L.	4.567	4.527
2	*Chenopodium album* L.	14.807	9.079
3	*Fallopia convolvulus*	3.220	2.435
4	*Fumaria parviflora*	1.750	1.476
5	*Orobanche* spp.	5.293	4.125
6	*Sinapis arvensis* L.	4.302	3.487
7	*Solanum lycopersicum* L.	6.740	4.915

Weed experts have examined the labels, compared them, and confirmed their accuracy and consistency. The dataset obtained in this study is considered ideal for identifying multiple weed species in tomato production systems in Türkiye. It reflects the true diversity and variability of agriculture under different environmental conditions.

Data augmentation was performed to make the dataset more stable and to enable the model to work with high accuracy in many cases. These included horizontal, vertical flipping and horizontal, vertical scrolling (geometric transformations). Python was used for model training, and Visual Studio Code (VSCode) was used for writing and running the code.

**Figure 4:**
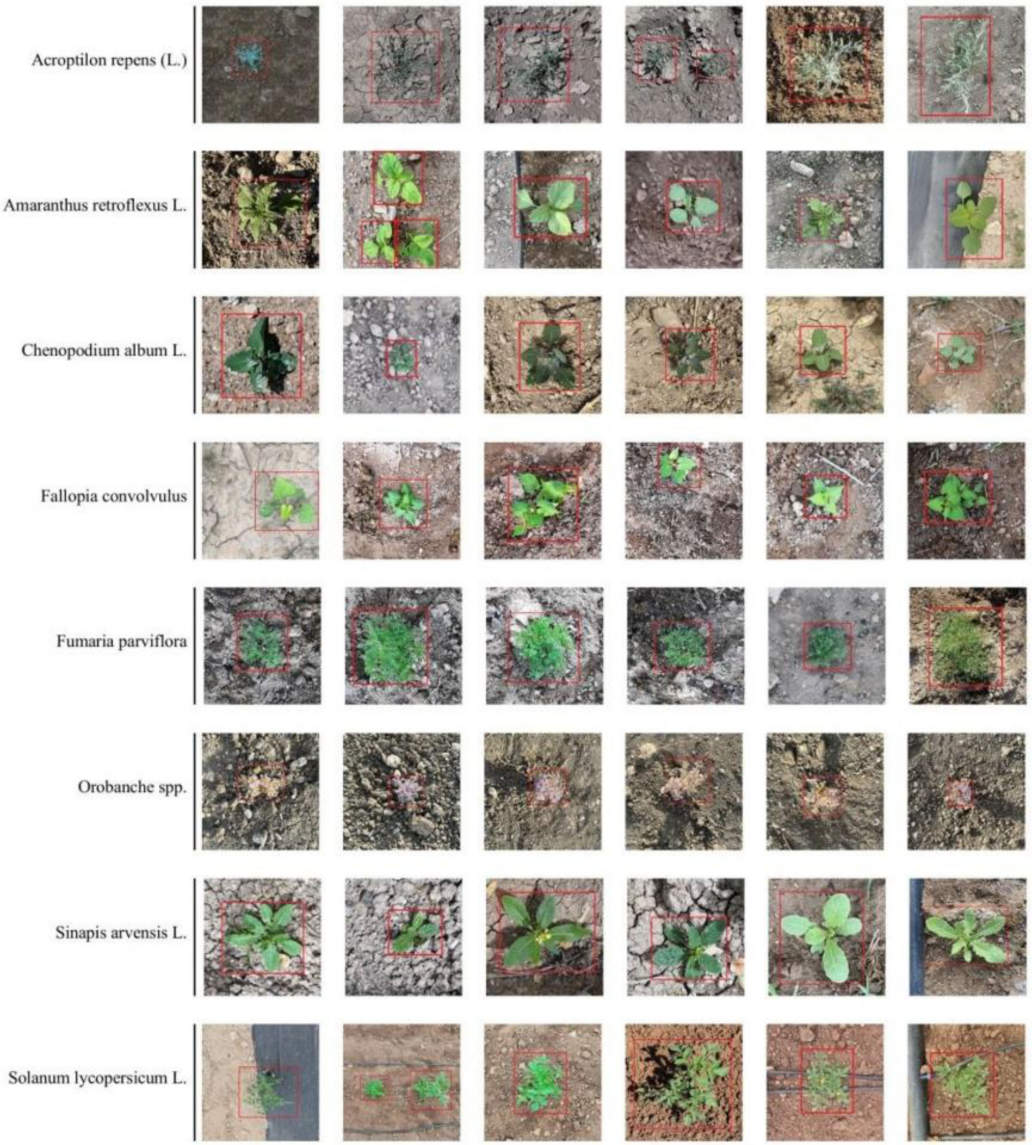
Sample images taken from the TomatoWeedDet dataset. Each row indicates the bounding boxes around randomly selected samples from eight classes (seven weed species and tomatoes).

**Figure 4** shows example annotated images where only one weed class is highlighted in each row. Differences exist between and within weed classes in terms of plant morphology/color, soil background, and field light conditions; these differences are desirable characteristics for creating models that are robust to imaging conditions. This dataset addresses a significant gap in the literature by adding previously unstudied taxa to the list of commonly found weed species in fields. These 7 weeds are frequently encountered in Turkish agriculture. Finally, the inclusion of data under real agricultural field conditions, taking into account natural damage such as soil type, light angle, and plant shape, adds significant originality to weed detection.

### YOLO object detectors

Object detection algorithms using deep learning consist of two parts: the backbone and the head (Ren et al., 2017). The backbone performs feature extraction from high-dimensional image inputs and is generally built upon models pre-trained on ImageNet. The head, on the other hand, is responsible for object classification and bounding box regression. Depending on the presence of a separate region proposal module, object detectors are categorized into two-stage and one-stage architectures (Carranza-García et al., 2020). All YOLO types are one-stage detectors that perform both classification and localization.

**Figure 5:**
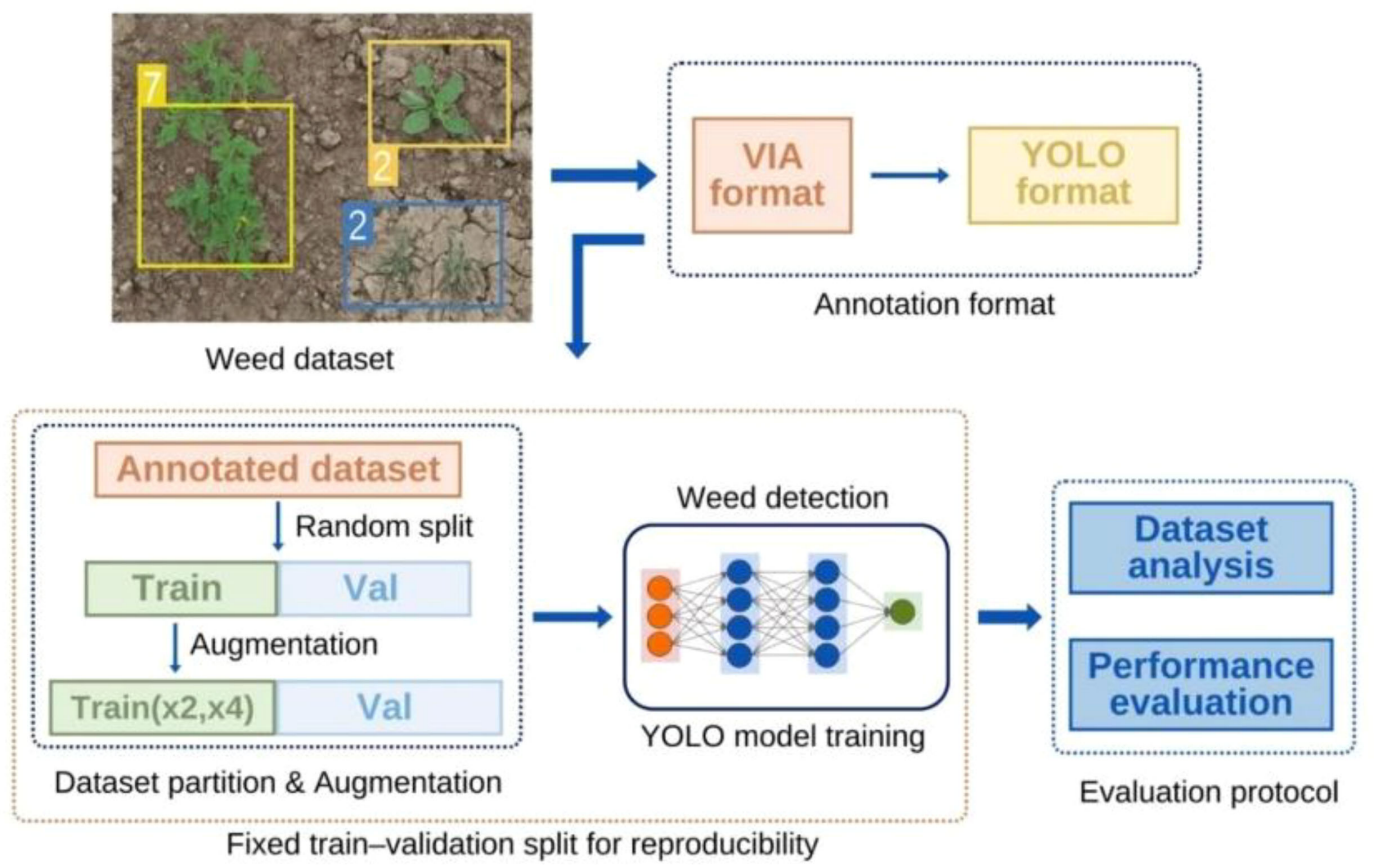
The proposed pipeline of weed detection by YOLO object detectors.

**Figure 5** illustrates the general workflow of the weed detection algorithm used. Image descriptions, generated in JSON format, were converted to YOLO-compatible text files to ensure compatibility with all YOLO architectures. The labeled dataset was divided into training and validation subsets in a controlled manner to prevent data leakage and ensure experimental consistency. A fixed data splitting approach was preferred to maintain consistency and reproducibility across all experiments.

YOLOv3-Tiny (Huang et al., 2018) is a lightweight network architecture derived from YOLOv3 for real-time object detection. It adopts Darknet-19 as backbone and down samples the layers with high resolutions to a computation-effective one on resource-limited embedded devices. Although highly successful, YOLOv3-Tiny is also adopted for light network and tiny-object detection task (Calì et al., 2025).

The small but fast YOLOv5s (Jocher et al., 2020), the smallest of YOLOv5 series has anchor-box optimization and auto learning rate schedules for successful training. Based on the modular design and lightweight structures of x-vision, it’s quite convenient to be used in embedded devices on edge side or mobile phone. Modify while YOLOv5s can handle small object detection very well, in contrast with the previous (Deng et al., 2023).

YOLOv8 from Ultralytics is the next evolution of YOLO design. Anchors: Our method uses an anchor-free architecture with a decoupled head which can process classification and localization more flexibly (Li et al., 2025). We tested four different YOLOv8 models which differ in computational cost in this work:

- YOLOv8n (nano): It becomes the fastest and lightest structure particularly dedicated to low-powered devices (Shi et al., 2025).
- YOLOv8s (small): Offers low computational cost with sufficient accuracy for real-time applications.
- YOLOv8m (medium): Delivers high accuracy at low computational cost for real-time scenarios.
- YOLOv8l (large): Includes more parameters and results in better accuracy, desirable for precision-demanding tasks.

The main reasons are that YOLOv8 models have more advanced data augmentation strategies and enriched path-aggregation neck designs, enabling competitive small-object detection performance (Qiu et al., 2025; Ryu et al., 2025).

YOLOv10n family was presented in 2024. It is built with advanced architectural changes to deliver the highest precision, and performance when using these precision formats. This method includes the multi-scale feature fusion and the context-aware backbone, which are more powerful in representing information (Cai et al., 2024). When the parameter size is scaled down, YOLOv10n can produce more accurate detections than its counterpart YOLOv8n (Wan and Ming, 2025).

In the present study, seven YOLO models were utilized for multiclass weed detection in tomato fields. Each model was trained under customized and uniform experimental settings sourced from the open-source GitHub repository given in **Table 2** by its developers.

**Table 2:** YOLO models employed and links to open source access sources.

Index	YOLO model	URL (open source repository)	Reference
1	*YOLOv3-Tiny*	https://github.com/pjreddie/darknet	Redmon and Farhadi (2018)
2	*YOLOv5s*	https://github.com/ultralytics/yolov5	Jocher et al. (2020)
3	*YOLOv8n*	https://github.com/ultralytics/ultralytics	Li et al. (2025)
4	*YOLOv8s*	https://github.com/ultralytics/ultralytics	Li et al. (2025)
5	*YOLOv8m*	https://github.com/ultralytics/ultralytics	Li et al. (2025)
6	*YOLOv8l*	https://github.com/ultralytics/ultralytics	Li et al. (2025)
7	*YOLOv10n*	https://github.com/THU-MIG/yolov10	Cai et al. (2024)

The main performance metrics which are often utilized for the evaluation of object detectors are Precision (P), Recall (R), and mean Average Precision (mAP).

- Recall (R): It measures the proportion of actual objects that were identified correctly. The recall value is higher if the ability to capture all objects of relevance without omission. This statistic is particularly relevant in mission critical fields like agriculture, health care and security where the failure to detect a true event has significant consequences (Zhao et al., 2019).
- Precision (P): Precision measures the true ratio of the detected objects. A low precision indicates that the model is not reliable because of false-positive predictions. For real-time scenarios where too many false alarms could cause unacceptable impacts, perfect precision is preferable (Padilla et al., 2021).
- mean Average Precision (mAP): mAP depicts average precision averaged over all object classes and is usually also at certain IoU thresholds. It is also the standard evaluation criterion in object detection, such as PASCAL VOC and COCO (Everingham et al., 2015) and offers a complete depiction of model capability. For instance, as a result of substantial architectural improvements, the YOLOv8l model achieves an average mAP of 37% on COCO whereas the YOLOv5s only reaches an average mAP of up to 51% (Jocher et al., 2020; Li et al., 2025) (Jocher et al., 2020; Li et al., 2025). In the present research, across the abovementioned metrics, we found that seven variants of YOLO performed would differently. Light models like YOLOv3-Tiny and YOLOv5s had lower mAP but could achieve higher speed, which was desirable for embedded/mobile applications. On the other hand, YOLOv8l and YOLOv10n are large models that achieved higher mAP values and more balanced precision-recall trade-offs, which would be more suitable for applications with demanding precision under non-uniform field conditions (Cai et al., 2024; Shi et al., 2025).

### Experimentation

The annotation files of the dataset used in this study were initially created in.txt format and were converted to a YOLO-compatible structure using a custom-developed Python script. During this conversion, each class’s bounding box coordinates were rewritten in the normalized format required by YOLO algorithms. The bounding boxes annotations dataset was carefully divided into training (70%), validation (20%) and test (10%) subsets to prevent data leakage. A fixed data split, rather than K-fold cross-validation, was used to maintain independence between training and validation sets and ensure fair and consistent model comparison.

Model training was conducted with the PyTorch deep learning framework. The hyperparameters in training were given as: epochs=200, image size=416×416, batch size=16, workers=2, patience=200. All YOLO models were trained with same hyperparameter setting to ensure fairness of comparison. In the course of training, model performance was evaluated on the validation set at every epoch and saved the weights corresponding to the best evaluation. This best model weights were then finally used for inferencing, and the very final weights of each partially trained models at the end, too were evaluated as a comparison.

All the training experiments are implemented on a machine with an NVIDIA RTX A2000 GPU (6 GB VRAM, 3,328 CUDA cores, Ampere architecture). In order to avoid a GPU memory crash, parameters of batch size and image resolution were manually adjusted according to hardware restrictions.

Data augmentation were enforced by hand to improve the generalization of the model, and reduce overfitting. All images were subjected to elementary geometric operations (flipping, left-right rotation, and 90° rotations) These augmentations were performed in preprocessing (prior to training) rather than on-the-fly during training, meaning that all models trained with an equally augmented dataset. Augmented images were only included in the training and validation datasets, and no augmentation process was applied to the test dataset. This was intended to minimize potential data leakage. Differently from existing works which adopted augmentation on certain YOLO variants, all the models are trained with a uniform set of augmentations for experimental fairness.

To provide a fair comparison that clearly shows how the different YOLO architectures compare, each model was trained and tested on the same dataset, with the same parameters and data augmentation process. Results were reported in terms of mAP, P and R, as defined and justified in the next sections.

### Dataset partitioning and leakage prevention

The dataset was divided into training, validation, and testing subsets at percentages of 70%, 20%, and 10%, respectively. Before performing the data splitting process, all images were manually reviewed, and consecutive or highly similar frames, particularly those that could occur in drone footage, were removed from the dataset. Furthermore, the dataset was not obtained via drone or phone video recording. All data was obtained by manually photographing individual images. The resulting dataset contains images of weeds from different angles and distances. This approach aimed to reduce the risk of data leakage due to highly similar images that could arise from consecutive video frames. Thus, it aimed to prevent potential spatial data leakage between the training, validation, and testing sets.

The images were collected on different dates, at different times of day, and under different environmental conditions. Therefore, the dataset naturally contains temporal variations. During the data splitting process, care was taken to ensure that highly similar images from the same area did not end up in different datasets. This approach aims to improve the ability of models to generalize to different field conditions, rather than simply memorizing specific image samples.

### Training parameters and implementation details

All models were trained using the same hyperparameters. AdamW was chosen as the optimizer during the training process. The initial learning rate was set at 0.01 and gradually decreased throughout the training process, reaching approximately 0.0001 in the final epochs. A confidence threshold of 0.25 and an IoU threshold of 0.7 were used in the model evaluation phase. Training parameters and implementation details are shown in **Table 3**.

**Table 3:** Training parameters and implementation details.

Parameter	Value
Optimizer	AdamW
Initial Learning Rate	0.01
Final Learning Rate	0.0001
Confidence Threshold	0.25
IoU Threshold	0.7
Augmentation	Mosaic, HSV, Flip
Input Size	416×416
Framework	Ultralytics YOLO

Mosaic augmentation, HSV-based color transformations, and flipping operations were applied during the data augmentation process. The training parameters and augmentation strategies used were applied identically to all models to ensure experimental consistency. The hyperparameters used in this study were determined based on the recommended default configurations of the Ultralytics YOLO framework. In this study, a resolution of 416 × 416 was chosen as the input image size. This size was selected to provide a balance between computational cost and detection performance. It offers lower memory consumption and is considered a suitable solution for embedded system scenarios due to its faster processing time.

## Results

The comparison of YOLO models for weed detection on tomato fields emphasized three main aspects: accuracy of detection, computational requirement, and inference time. The introduced the model is evaluated with P, R, mAP@0.5 and mAP@[0.5:0.95] that are consistently used as standard metrics in object detection literature (Everingham et al., 2015; Padilla et al., 2021) (Everingham et al., 2015; Padilla et al., 2021). This provides evidence that the model being developed gives consistent results at various levels of detection resolution.

### Model performances

The average validation performance of the trained models is given in **Table 4**. The YOLOv8l model achieved the highest mAP@0.5 value at 97.8% and the highest mAP@[0.5:0.95] score at 93.8%.

**Table 4:** Performance results of seven YOLO algorithms in weed detection.

Model	P	R	mAP@0.5	mAP@[0.5:0.95]
YOLOv3-Tiny	96.2 ± 2.06	93.8 ± 0.96	95.4 ± 2.51	87.6 ± 1.20
YOLOv5s	97.3 ± 1.53	94.2 ± 0.81	95.3 ± 2.62	91.1 ± 0.53
YOLOv8s	97.7 ± 0.95	94.1 ± 1.12	96.2 ± 1.71	91.6 ± 0.88
YOLOv8n	96.7 ± 2.02	93.8 ± 1.18	96.1 ± 1.98	89.6 ± 0.81
YOLOv8m	98.2 ± 0.77	94.7 ± 0.98	97.1 ± 0.97	92.9 ± 0.68
YOLOv8l	98.0 ± 0.78	95.6 ± 0.69	97.8 ± 0.61	93.8 ± 0.84
YOLOv10n	96.7 ± 1.43	91.4 ± 2.89	96.2 ± 0.88	88.8 ± 1.67

This was followed by YOLOv8m (97.1%), YOLOv8s (96.2%), and YOLOv10n (96.2%). The lightweight variant, YOLOv3-Tiny have mAP@[0.5:0.95] values of 87.6%. They appear to achieve slightly lower, but satisfactory, performance levels.

The same trend is observed for P and R. The YOLOv8l model is close to the optimum balance, achieving 98.0% P and 95.6% R. This indicates that the model maintains high accuracy and minimum false positive rates. The YOLOv8m and YOLOv8s models demonstrate superior performance. The YOLOv5s and YOLOv3-Tiny models appear to have moderate performance with slightly lower recall values.

### Comparative evaluation of models

**Figure 6:**
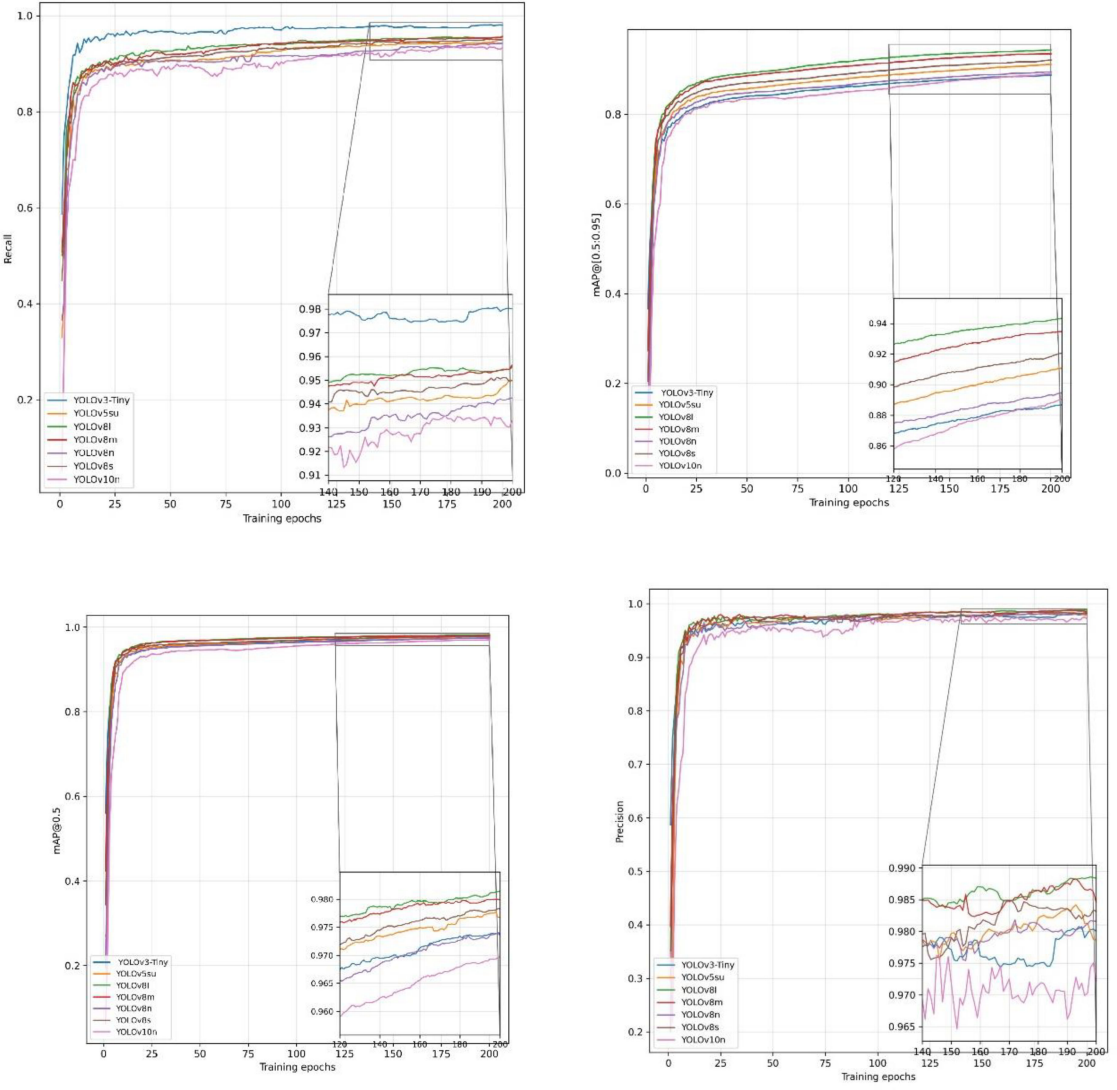
P and R training curves over 200 epochs for seven YOLO-based models evaluated for multi-class weed detection. The graphs provide an enlarged view of the final training phases to highlight convergence behavior and performance differences between the models.

Training performance of all YOLO models on 200 epochs is shown in **Figure 6**, mAP@[0.5:0.95], precision, and recall. All of the models trained quickly and converged across the first 25 epochs, from which point forward there was minimal effective learning. YOLOv3-Tiny model had low parameter number and high processing speed, but it underperformed compared to models with more complex architectures resulting 90.07% for mAP@[0.5:0.95]. This performance degradation can be partially attributed to its limited backbone network (Darknet-19) which prevents it from being discriminative enough for small weed instances in cluttered field scenes (Redmon and Farhadi, 2018).

YOLOv8l achieved the best overall performance with 98.2% mAP@0.5 and 94.4% mAP@[0.5:0.95], achieving more than 98% precision and 95% recall. Both YOLOv8m and YOLOv5s had competitive performances, lighter models like YOLOv3-Tiny, and YOLOv10n showed inferior performance at faster convergence. By contrast, we observed ~4% mAP@[0.5:0.95] improvement of the YOLOv5s model over YOLOv3-Tiny, mainly owing to its anchor-box refinement and PyTorch-based dynamic learning techniques. These architectural innovations allowed YOLOv5s to balance accuracy and computational cost better. However, the YOLOv8 family put a great focus on both training efficiency and detection accuracy with its anchor-free reference framework and upgrade decouple head (Shi et al., 2025. These advancements allow YOLOv8 models to achieve enhanced localization and classification performance for multi-scale objects amidst cluttered or heterogeneous agricultural backgrounds.

In conclusion, even with a reduced number of parameters, YOLOv10n is seen to achieve a good result with an mAP@[0.5:0.95] of 88.8%. This is due to the integration of multi-level feature fusion and context-sensitive attention blocks into its architecture. It shows that it significantly improves model robustness, especially for low-resolution images (Cai et al., 2024).

All models exhibited stable accuracy values ​​after approximately 200 epochs, and the learning curves stabilized consistently. YOLOv8l demonstrates superior accuracy, stability, and generalization capacity. It has proven itself as the most efficient model for multi-class weed detection in tomato fields.

### Comparison with literature and discussion

In this study, when the results obtained are evaluated, it is seen that high performance values, acceptable among multi-class weed detection studies found in the literature, have been achieved.

For instance:

- In the YOLOWeeds study by Dang et al. (2023), the best-performing model, Scaled-YOLOv4, achieved an mAP@[0.5:0.95] of 89.5%.
- The RLRD-YOLOv8 model proposed by Li et al. (2025) demonstrated strong performance, reaching 91.3% mAP@[0.5:0.95].
- Similarly, the OMB-YOLO-tiny model developed by Shi et al. (2025) achieved 89.7% mAP@[0.5:0.95] in detecting small-sized objects.

In this context, the YOLOv8l model in the present study reached an mAP@[0.5:0.95] of 93.8%, which represents a competitive accuracy score compared with related studies. The ability to achieve high P (≈98%) and R (≈95%) simultaneously illustrates that the model can effectively minimize false negatives, i.e., guarantee that correct classification is made. The balanced behavior presented herein indicates that the YOLOv8l and YOLOv8m architectures are a proper compromise between high precision and applicability to real agricultural field. They are found to be the most appropriate models for early weed detection in tomato cultivation.

### Model complexity and real-time applicability

The quantity of model parameters, FLOPs and inference time are important considerations that directly affect the model choice, especially in agricultural robots and embedded vision systems (Zhao et al., 2019). The YOLOv3-Tiny and YOLOv10n models, with their lightweight network architectures, can deliver the fastest FPS performance, making them suitable for real-time applications. The model YOLOv8l has even more complex architecture; however, having a well-tuned computational design allows for an inference speed in real-time about 20–25 FPS.

It has observed that the YOLOv8l model can be applied effectively to the field-based drone imaging systems and also mobile device applications, implying its flexibility and convenience for real-world agricultural agriculture operations (Li et al., 2025; Qiu et al., 2025) (Li et al., 2025; Qiu et al., 2025).

To more accurately assess the practical applicability of the proposed models under real-time agricultural scenarios, computational performance comparison analyses were also conducted. FPS, inference latency, FLOPs, model size, and GPU utilization values ​​were evaluated comparatively under the same hardware conditions.

**Table 5** presents the computational complexity and real-time applicability results of the evaluated YOLO architectures. The results show that lightweight models such as YOLOv3-Tiny, YOLOv5s, and YOLOv8n achieve significantly higher FPS values ​​and lower inference latency due to their lower computational complexity. Larger models such as YOLOv8m and YOLOv8l provided higher detection accuracy at the expense of increased FLOPs, model size, and processing latency.

**Table 5:** Computational complexity and real-time performance comparison of YOLO architectures.

Model	FLOPs	Model Size	FPS	Latency (ms)	GPU Utilization
YOLOv3-Tiny	2.4 G	6 MB	220	4.5	50%
YOLOv5s	6.1 G	9 MB	160	6.2	60%
YOLOv8n	8.9 G	7 MB	145	6.9	68%
YOLOv10n	7.5 G	6 MB	170	5.8	65%
YOLOv8s	22 G	22 MB	100	10	80%
YOLOv8m	54 G	55 MB	60	16.5	92%
YOLOv8l	88 G	92 MB	33	30	98%

These findings demonstrate a balance between detection performance and computational efficiency. Larger architectures offer improved feature extraction capability and higher detection accuracy in complex field conditions. Lightweight models may be more suitable for embedded systems, edge computing platforms, and real-time precision agriculture applications where computational resources are limited.

### Extended dataset and performance comparison

In this research, the performance of seven YOLO-based models and New proposed model: YOLO(v10n) were compared with recent deep learning-based weed detection studies available in the literature. The performance was evaluated in terms of P, R, mAP@0.5, mAP@[0.5:0.95], size of the dataset, number of classes kernels learnt, image capturing settings and devices used for training and testing the models.

According to the results, the YOLOv8l model achieved the highest overall performance, with P = 0.98, R = 0.956, mAP@0.5 = 0.978, and mAP@[0.5:0.95]=0.938. This accuracy level is considered competitive compared with related agronomy-related object detection studies, especially for detecting small and clustered weed instances.

The results and related state-of-the-art research are compared on the datasets in **Table 6**. This table shows that the proposed dataset and models are in a competitive range in terms of scale, diversity, and true-field realism.

**Table 6:** Comparison of deep learning-based weed detection studies.

Study	Model	Dataset Size	No of classes	P	R	mAP@0.5	mAP@[0.5:0.95]	Platform
This study	YOLOv8l	32.607	8	0.98	0.956	0.978	0.938	Drone + Phone
Skacev et al., 2020	CNN	1.500	5	N/A	N/A	N/A	N/A	Phone
Das et al. (2025)	YOLOv7	9.249	2	0.87	0.78	0.88	0.5	Drone
Li et al. (2025)	PD-YOLO	5.648	12	0.943	0.87	0.95	0.883	Phone + Camera
Sudars et al. (2020)	N/A	1.118	8	N/A	N/A	N/A	N/A	Camera
Dang et al. (2023)	Scaled-YOLOv4	5.648	12	0.961	0.936	0.951	0.895	Phone + Camera
Olsen et al. (2019)	ResNet-50	17.509	8	0.957	0.951	N/A	N/A	Camera
Li et al. (2025)	RLRD-YOLOv8	N/A	10	N/A	N/A	0.951	0.628	UAV
Jiang et al. (2020)	GCN-ResNet-101	6,000	9	0.983	0.996	N/A	N/A	Camera
Wang et al. (2022)	TIA-YOLOv5	4,500	N/A	0.91	0.81	0.87	N/A	Camera
Zhou et al. (2025)	YOLO-ACE	5,648	12	0.961	0.896	0.953	0.895	Phone + Camera
Tao and Wei (2024)	STBNA (YOLOv5 Based)	5,000	4	0.644	0.881	0.908	N/A	N/A

### Per-class performance evaluation

Class-based evaluation metrics and confusion matrix analyses were performed for all weed species and tomato plants included in the proposed benchmark dataset. Precision, recall, and mAP values ​​indicate that classes are distinguishable under heterogeneous real-field conditions.

**Figure 7:**
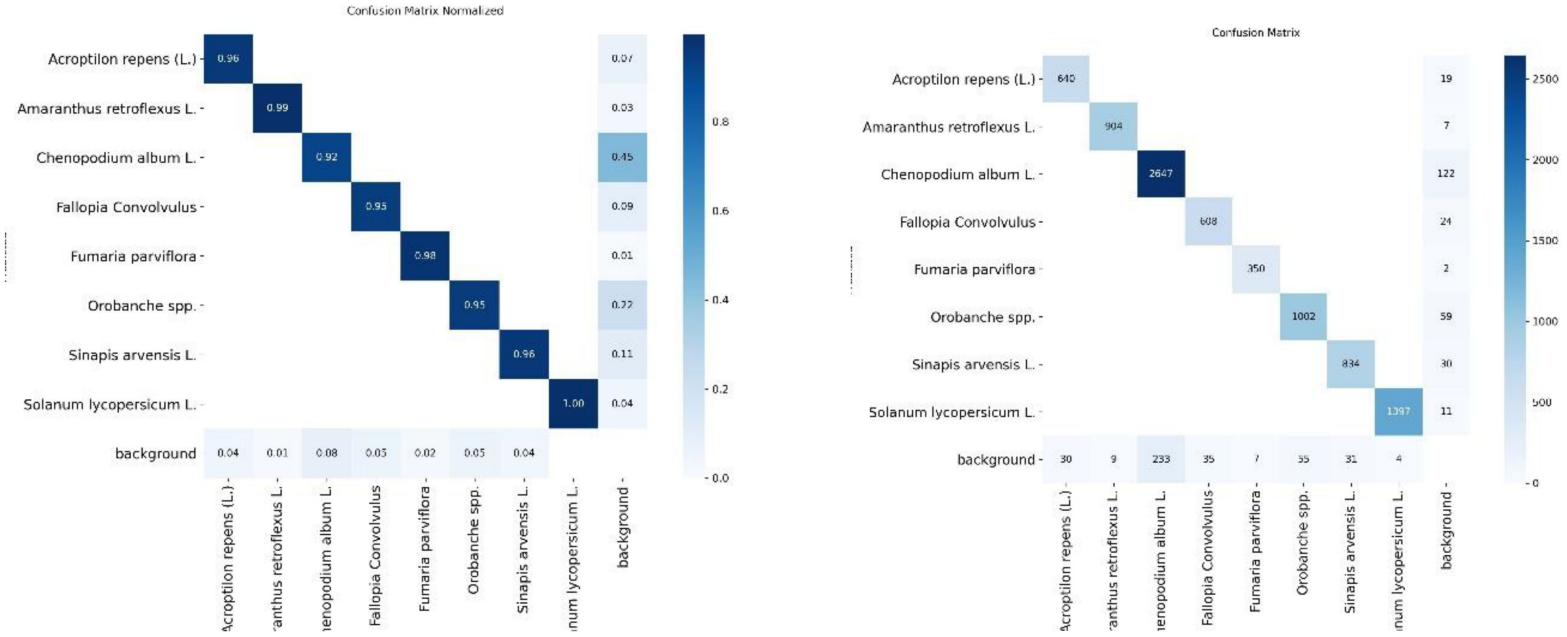
Normalized confusion matrix and confusion matrix of the YOLOv8l model for multi-class weed detection.

The normalized confusion matrix also reveals that some classes exhibit relatively higher background confusion under challenging environmental conditions. Specifically, Chenopodium album L. showed relatively higher confusion due to soil similarity, shade effects, and background regions observed in certain field images. Solanum lycopersicum L. achieved high detection performance due to its relatively distinct morphological features and stronger representation in the dataset. The confusion matrix results presented in **Figure 7** illustrate the confusion pattern between the evaluated weed species and tomato plants.

**Figure 8:**
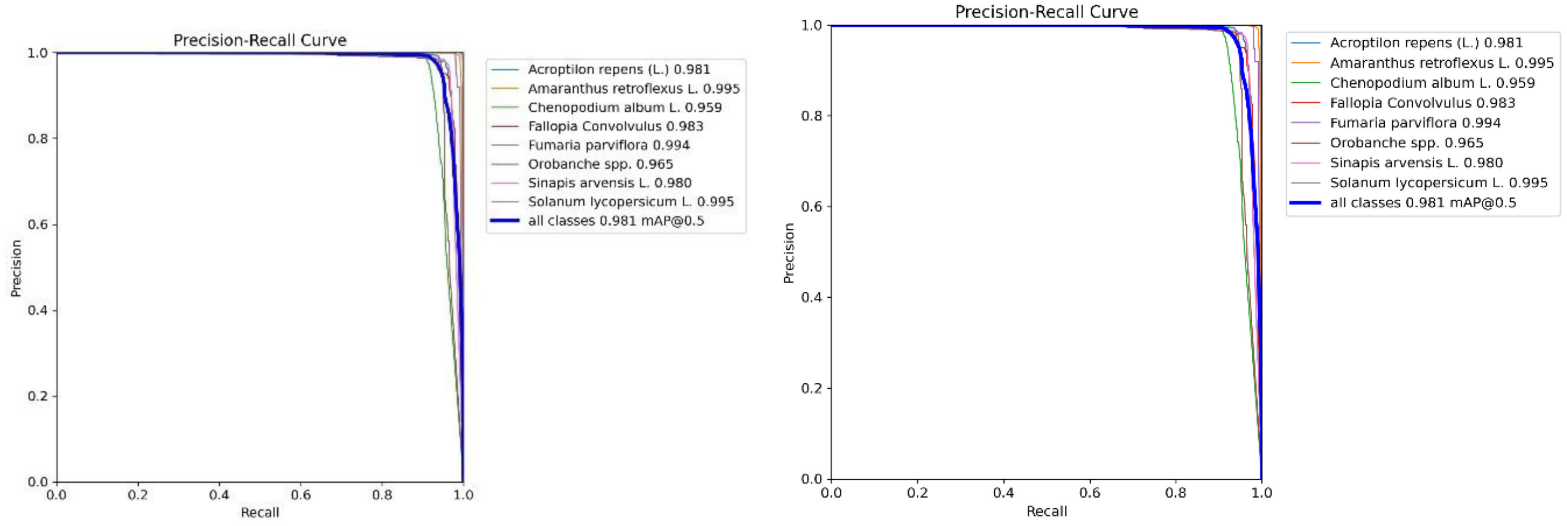
The per-class precision-recall curves of the evaluated weed species and tomato plants.

The per-class precision-recall evaluation revealed that most weed species and tomato plants included in the dataset exhibited generally consistent detection performance. Classes such as Amaranthus retroflexus L., Fumaria parviflora, and Solanum lycopersicum L. displayed relatively stable precision-recall characteristics throughout the evaluation process. Chenopodium album L. and Orobanche spp. showed relatively lower performance values. This is thought to be related to smaller object structures, complex background conditions, and heterogeneous field environments. The confusion matrix results presented in **Figure 7** illustrate the confusion pattern between the evaluated weed species and tomato plants. As shown in **Figure 8**, the precision-recall curves demonstrate consistent performance across the classes included in the dataset.

Class-based analyses demonstrate that the proposed dataset enables multi-class weed detection under real-world agricultural conditions, highlighting the challenges associated with visually complex field environments.

### Failure case analysis

**Figure 9:**
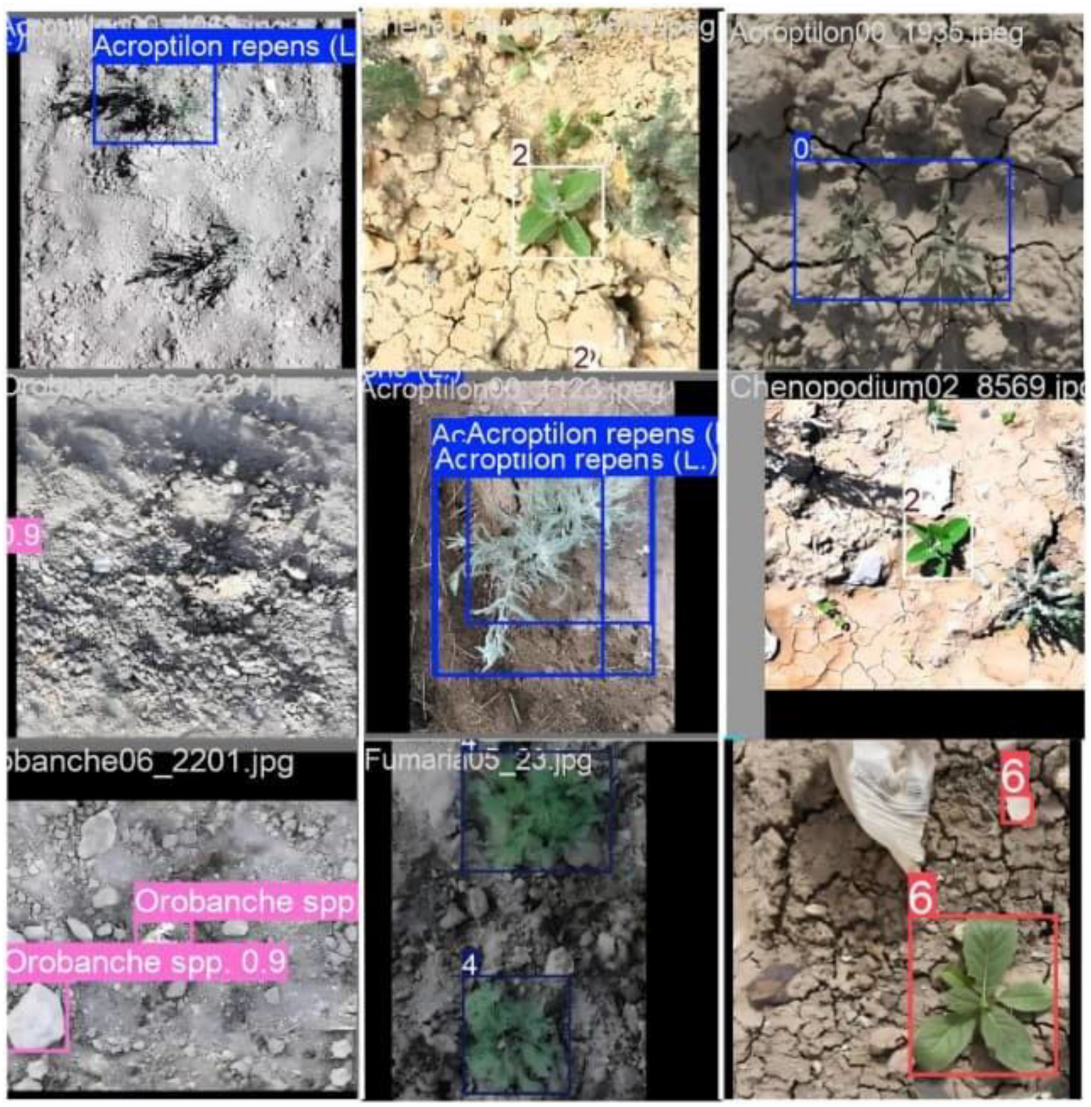
Representative failure cases under challenging real-field conditions.

The proposed benchmark model achieved successful detection performance in most classes. However, failure cases were observed under challenging field conditions. Representative examples are presented in **Figure 9**. Detection failures occurred in data containing small weed samples, overlapping vegetation structures, low visual contrast between weeds and soil background, shadow effects, and partially obscured plants.

In dry soil conditions and uneven lighting, detection was challenging in weeds where soil and weed color were similar. Similarly, overlapping plant structures and environmental artifacts such as mud, shadows, and uneven soil textures occasionally reduced detection confidence. These observations demonstrate that highly heterogeneous agricultural environments are challenging, even for advanced deep learning-based object detection models.

The presented failure cases provide additional insights into the limitations of the evaluated models and highlight the importance of improving robustness for real-world precision agriculture applications. Future studies with lightweight architectures optimized for larger cross-field datasets and challenging field conditions could further improve detection performance.

### Dataset evaluation and key differences

Meaning While data derived from a single imaging source are more challenging to apply across application fields, an extensive review demonstrates that almost all publicly accessible weed detection databases have been captured from either a ground-based camera or a UAS. However, datasets containing examples selected from a homogeneous source tend to have consistent and visually similar images, whereas there is time diversity in terms of geometric and environmental appearance that may hinder the robustness of trained models in outdoor conditions when moving toward real-world consistency. In contrast, we have constructed the dataset in this work through utilizing combination of acquisition platforms, two different mobile cameras and a drone system that can cover close-range and aerial shots. Also, multi-source setting make the dataset more realistic and inclusive to make the model generalization better for various kinds of lightening, soil textures and view angles that are faced by farmers in real life.

In this study, the proposed data set consists of various samples from different time periods for three distinct regions of Türkiye, namely Balıkesir, Ankara and Aksaray. This diversity has improved this model robustness to light intensity, shadow distribution, soil reflectance and plant texture changes. By using multiple image acquisition platforms such as two smartphones and a DJI Mavic 3 Pro drone, the multi-perspective data of various weeds were collected such that each model could be capable of recognizing weeds at both proximal and aerial views.

The diversity in soil type (e.g., clay, sandy, and organic-rich soils) also provided natural variation of color and contrast that adds to the dataset’s ability to capture real-world agricultural scenarios. Thus, the dataset proposed in this paper has a very realistic and field-applicable architecture, which is closer to real agricultural scenes than prior works.

### Comparative interpretation of the results

The high detection performance (mAP@[0.5:0.95] = 0.938) obtained stems not only from the efficiency of YOLOv8 architecture but also due to the dataset with varied and multi-source images built in actual field conditions. The vast majority of available datasets for weed detection study are created from homogeneous conditions and a single point of view, which can compromise to varying degrees the generalization ability of the model under different real scenarios. By contrast, the database considered in this paper includes images taken with different systems (drone and dual mobile camera), which makes variations in terms of: illumination changes, texture trends and appearance of the plant. This variety has substantially increased the capacity of the model to generalize over ecologically complex field conditions. Thus, both evaluation metrics mAP@0.5 (0.978) mAP@[0.5:0.95] (0.938) also remained high, indicative of the approaches robustness and practical use in real-life situations.

This stability can be attributed to the following:

- Real-field diversity; lighting, soil color and weather,
- Various image sources, aerial and ground viewpoints,
- A relatively large bounding boxes annotations dataset with 32,607 images and 44,165 labels,
- Manual annotation, which in turn reduced the label noise and miss-class,

Accordingly, the models developed in our study achieved statistically generalizable accuracy results for weed detection. Although similar level of accuracy has been reported in other deep learning applications, the diversity of data sets, extensive annotation efforts, and considerations on environmental differences differentiate this work. Furthermore, two weed species not been examined by the deep learning-based detection methods, are included in this study dataset, evidencing its novelty and being part of an initial stage process for practical field-oriented weed detection. The comprehensive nature of the dataset and the robustness of the applied methodology provide a valuable basis for future advancements in precision agriculture and intelligent field automation.

## Discussion

Deep learning and computer vision are rapidly developing. There is insufficient research in the literature on multi-class weed detection and localization. The main reason for this deficiency is the lack of sufficiently large and multi-class datasets obtained from real agricultural fields. Vision-based weed recognition systems can generally detect only a small number of weed species. This study includes seven weed species and tomato plants. It systematically compares seven YOLO architectures run on the same hyperparameter. The results of the YOLOv8l algorithm, with mAP@0.5 = 97.8% and mAP@[0.5:0.95]=93.8%, demonstrated promising performance in comparison with related literature. The study utilized YOLO architectures, which are widely used and accepted in the literature. Evaluation of newer generation models and architectural innovations are planned for future studies. The proposed benchmark framework can contribute to precision spraying systems by providing weed detection with class awareness. Detecting weed species will help reduce unnecessary herbicide use.

Lightweight YOLO architectures have demonstrated computational capabilities suitable for drone-based monitoring systems and edge computing-supported agricultural applications. Their rapid inference capability and low computational requirements can provide practical advantages in real-time field monitoring applications. However, further optimization studies are needed before direct use in fully autonomous agricultural systems.

The most important distinguishing feature of this study is that the proposed dataset provides real-world field conditions. Natural vegetation images (rain, mud, hail marks, faded plants, etc.) and different soil conditions (clay, sandy, and organic matter-rich soils) were obtained in three provinces of Türkiye (Balıkesir, Ankara, and Aksaray) at different times of day (morning, noon, and afternoon). Environmental diversity significantly enhances the generalization ability of the models. Validation studies using data from different countries or independent datasets will support a more comprehensive assessment of the model’s generalization capacity. A few degraded images due to rain, hail, mud and shadows are also included in the dataset for simulating challenging conditions of field. The present study is unique compared to other work in this regard because it specifically takes into account the role of natural environmental variability, as opposed to using only homogenous/controlled data sets. Factors such as high light variations in real-field conditions, overlapping weeds, and small object sizes can make detection performance difficult in some cases. Utilizing the drone and two types of smartphone cameras as image sources enabled the model to detect objects at various resolutions and orientations. The combined use of drone and smartphone imagery aims to increase the visual diversity of the dataset under different viewing angles and sensor characteristics, and to partially improve the robustness of the benchmark structure. The aim of this study is not to propose architectural innovations, but rather to create a highly diverse dataset reflecting real-world field conditions and a standardized benchmark infrastructure. Such multi-source integration permits to keep the accuracy of the algorithm high when compared with data coming from multiple devices or environmental situations (Feng et al., 2021).

### Limitations and future work

The proposed dataset includes images collected from different agricultural regions and environmental conditions. Some limitations must be considered when evaluating the results of this study. The dataset was created from data obtained from three different provinces of Türkiye under varying light conditions, soil structures, weather conditions, and image acquisition angles. The aim was to increase environmental diversity and improve the robustness of the models in real agricultural conditions. The dataset was collected using drones and phones. Although collected from three different provinces and different fields in Türkiye, the dataset still represents a limited geographical region and climate profile.

A significant limitation concerns the generalization ability of the models across different seasons and plant development stages. The dataset generally includes visually distinguishable stages of weeds. Model performance may vary in different phenological stages, seasonal conditions, or agricultural practices. Different camera systems, sensor qualities, and image resolutions can also affect model performance in real field applications.

The proposed benchmark demonstrated high detection performance under real field conditions. Validation supporting the results using independent datasets from different countries or agricultural environments could not be performed. Therefore, future studies require a broader review of the benchmark to better assess the model’s transferability and generalization capacity.

The practical use of deep learning-based weed detection systems can also present various operational challenges. Changes in lighting, shadows, camera lens contamination, overlapping weeds, motion-induced image distortions, wind, and hardware limitations associated with embedded systems can negatively impact real-time detection performance. Future studies are expected to focus not only on expanding the dataset under different environmental conditions and seasons, but also on developing lightweight and efficient application strategies for drone-based and autonomous precision agriculture systems.

## Conclusion

Weed detection is one of the most critical parts in machine vision for precision agriculture, and it is also crucial as part of plant-specific site spraying systems. Central to effective weed control is the generation of large-scale, reliably-annotated datasets and trained supervised learning models.

The tomato cultivation scenario generated a large, field-specific dataset containing a total of 32,607 images and 44,165 labels across eight classes (seven weed species and tomato plants). This corresponds to an average of approximately 1.35 tagged objects per image. Two of these seven weed species are, to the best of our knowledge, underrepresented in the current deep learning-based weed detection literature. Seven YOLO-based deep learning architectures were systematically compared under identical hyperparameters. The performance of the model was measured by precision, recall, mAP@0.5, and mAP@[0.5:0.95] metrics. The YOLOv8l model achieved the highest overall accuracy, with P = 98.0%, R = 95.6%, mAP@0.5 = 97.8%, and mAP@[0.5:0.95]=93.8%. These results outperform most existing deep learning methods at weed detection and show the good generalization ability of the YOLOv8 model on multi-class sharp crop images taken under natural field conditions.

One of the main merits of this work is to set up a dataset that well represents in-field agricultural environments. Photos were taken in different regions (Balıkesir, Ankara Aksaray), at various times of day (morning–noon–afternoon), under different soil contents or reflection styles such as clayey sandy organic rich; and natural factors reflecting the light condition like rain, hail shadowy etc. The addition of drone and smartphone imagery made the data more diverse, helping the model to generalize better in the field. This study presents an accurate detection model and an artificial intelligence system in situations representing practical, field conditions.

The results demonstrate the potential benefit of automation in weed detection when applied to agriculture.

Objectives of future research will be:

- Develop mobile applications enabling real-time weed identification by farmers using smartphones and tablets,
- Integrate trained models into embedded systems and drone-based real-time detection platforms,
- Enable target-specific micro-dosing of herbicides based on the detected weed type and location,
- Reduce unnecessary pesticide use, thereby lowering input costs and environmental chemical load,
- Ultimately support the transition toward sustainable and eco-friendly smart farming systems.

Furthermore, future research will focus on expanding the dataset to include additional climatic regions, topographic variations, and plant growth stages. Model testing in real field conditions will be extensively conducted using embedded devices (NVIDIA Jetson, Raspberry Pi, etc.).After scaling properly, it is available to real-time on mobile/drone platforms. It is also practical and efficient, which is another advantage.

## Data Availability

The datasets presented in this study can be found in online repositories. The names of the repository/repositories and accession number(s) can be found in the article/supplementary material.

## References

[B1] Abid M. M. Mahmood T. Ashraf R. Faisal C. N. Ahmad H. Niaz A. A. (2024). Computationally intelligent real-time security surveillance system in the education sector using deep learning. PloS One 19, e0301908. doi: 10.1371/journal.pone.0301908 38990958 PMC11238971

[B2] Aggarwal C. C. (2023). Neural networks and deep learning (Cham: Springer International Publishing). doi: 10.1007/978-3-031-29642-0

[B3] Brandsæter L. O. Mangerud K. Andersson L. Børresen T. Brodal G. Melander B. (2020). Influence of mechanical weeding and fertilisation on perennial weeds, fungal diseases, soil structure and crop yield in organic spring cereals. Acta Agricult. Scandinavica. Sect. B. — Soil Plant Sci. 70, 318–332. doi: 10.1080/09064710.2020.1728371 37339054

[B4] Cai Z. Chen R. Wu Z. Xue W . (2024). YOLOv8n-FAWL: Object detection for autonomous driving using YOLOv8 network on edge devices. IEEE Access 12, 158376–158387. doi: 10.1109/ACCESS.2024.3480976 25079929

[B5] Calì R. Falaschetti L. Biagetti G. (2025). Optimized implementation of YOLOv3-Tiny for real-time image and video recognition on FPGA. Electronics 14, 3993. doi: 10.3390/electronics14203993 30654563

[B6] Carranza-García M. Torres-Mateo J. Lara-Benítez P. Garcia-Gutiérrez J. (2020). On the performance of one-stage and two-stage object detectors in autonomous vehicles using camera data. Remote Sens. 13, 89. doi: 10.3390/rs13010089 30654563

[B7] Cha Y. Choi W. Büyüköztürk O. (2017). Deep learning‐based crack damage detection using convolutional neural networks. Comput.-Aided. Civ. Infrastruct. Eng. 32, 361–378. doi: 10.1111/mice.12263 40046247

[B8] Ciccone F. Ceruti A. (2025). Real-time search and rescue with drones: A deep learning approach for small-object detection based on YOLO. Drones 9, 514. doi: 10.3390/drones9080514 30654563

[B9] Coulson A. V. Thomas W. H. Wang C. (2025). A comparative study of deep learning-based models for object detection in remote sensing imagery. Int. Arch. Photogramm. Remote Sens. Spatial. Inf. Sci. XLVIII-M-5–2024, 201–205. doi: 10.5194/isprs-archives-XLVIII-M-5-2024-201-2025

[B10] Dang F. Chen D. Lu Y. Li Z . (2023). YOLOWeeds: A novel benchmark of YOLO object detectors for multi-class weed detection in cotton production systems. Comput. Electron. Agric. 205, 107655. doi: 10.1016/j.compag.2023.107655 38826717

[B11] Darwin B. Dharmaraj P. Prince S. Popescu D. E. Hemanth D. J . (2021). Recognition of bloom/yield in crop images using deep learning models for smart agriculture: A review. Agronomy 11, 646. doi: 10.3390/agronomy11040646 30654563

[B12] Das A. Yang Y. Subburaj V. H. (2025). YOLOv7 for weed detection in cotton fields using UAV imagery. AgriEngineering 7, 313. doi: 10.3390/agriengineering7100313 30654563

[B13] Deng L. Bi L. Li H. Chen H. Duan X. Lou H. . (2023). Lightweight aerial image object detection algorithm based on improved YOLOv5s. Sci. Rep. 13, 7817. doi: 10.1038/s41598-023-34892-4 37188735 PMC10185568

[B14] Dorafshan S. Thomas R. J. Maguire M. (2018). Comparison of deep convolutional neural networks and edge detectors for image-based crack detection in concrete. Constr. Build. Mater. 186, 1031–1045. doi: 10.1016/j.conbuildmat.2018.08.011 38826717

[B15] Esteva A. Kuprel B. Novoa R. A. Ko J. Swetter S. M. Blau H. M. . (2017). Dermatologist-level classification of skin cancer with deep neural networks. Nature 542, 115–118. doi: 10.1038/nature21056 28117445 PMC8382232

[B16] Everingham M. Eslami S. M. A. Van Gool L. Williams C. K. I. Winn J. Zisserman A . (2015). The pascal visual object classes challenge: A retrospective. Int. J. Comput. Vision 111, 98–136. doi: 10.1007/s11263-014-0733-5 30311153

[B17] FAO (2024). FAOSTAT: Crops and Livestock Products - Tomatoes (Production Quantity, 2023). Rome: Food and Agriculture Organization of the United Nations. Available online at: https://www.fao.org/faostat/en/#data/QCL (Accessed April 20, 2026).

[B18] Feng Y. Hu J. Duan R. Chen Z. (2021). Credibility assessment method of sensor data based on multi-source heterogeneous information fusion. Sensors 21, 2542. doi: 10.3390/s21072542 33916389 PMC8038569

[B19] Foxx A. Varrientos G. Kramer A. T. (2024). Multigenerational invasive plant competition causes greater root than shoot trait shifts in a perennial grass. Plant Ecolog. 225, 961–972. doi: 10.1007/s11258-024-01446-1 30311153

[B20] Hassan J. Gomasta J. Ali L. Sultana S. N. Zubayer M. Islam M. S. . (2024). Transforming weeds to edible vegetables: An alternative sustainable and ecofriendly approach to weed management. Weed. Manage. - Global Strat. doi: 10.5772/intechopen.1004883

[B21] Huang R. Pedoeem J. Chen C. (2018). “ YOLO-LITE: A Real-Time Object Detection Algorithm Optimized for Non-GPU Computers.” In: 2018 IEEE International Conference on Big Data (Big Data). (Seattle, WA: IEEE), pp. 2503–2510. doi: 10.1109/BigData.2018.8621865

[B22] Jiang H. Zhang C. Qiao Y. Zhang Z. Zhang W. Song C. (2020). CNN feature based graph convolutional network for weed and crop recognition in smart farming. Comput. Electron. Agric. 174, 105450. doi: 10.1016/j.compag.2020.105450 38826717

[B23] Jocher G. Nishimura K. Mineeva T. Vilariño R . (2020). YOLOv5. (New York, NY: Ultralytics LLC).

[B24] Kamilaris A. Prenafeta-Boldú F. X. (2018). Deep learning in agriculture: A survey. Comput. Electron. Agric. 147, 70–90. doi: 10.1016/j.compag.2018.02.016 38826717

[B25] Khaffagy A. E. Mazrou Y. S. A. Morsy A. R. El-Mansoury M. A. M. El-Tokhy A. I. Hafez Y. . (2022). Impact of irrigation levels and weed control treatments on annual weeds, physiological traits and productivity of soybean under clay soil conditions. Agronomy 12, 1037. doi: 10.3390/agronomy12051037 30654563

[B26] Kim J. Joe I. (2025). Deep learning-based drone defense system for autonomous detection and mitigation of balloon-borne threats. Electronics 14, 1553. doi: 10.3390/electronics14081553 30654563

[B27] Li H. Li Y. Xiao L. Zhang Y. Cao L. Wu D . (2025). RLRD-YOLO: An improved YOLOv8 algorithm for small object detection from an unmanned aerial vehicle (UAV) perspective. Drones 9, 293. doi: 10.3390/drones9040293 30654563

[B28] Litjens G. Kooi T. Bejnordi B. E. Setio A. A. A. Ciompi F. Ghafoorian M. . (2017). A survey on deep learning in medical image analysis. Med. Img. Anal. 42, 60–88. doi: 10.1016/j.media.2017.07.005 28778026

[B29] Margaryan G. Singh A. Rajput V. D. Elshikh M. S. Rawat S. Ghazaryan K . (2025). Salinity tolerance and growth response of redroot pigweed (Amaranthus retroflexus L.): A comprehensive evaluation. PeerJ 13, e19717. doi: 10.7717/peerj.19717 40985027 PMC12450372

[B30] Maxwell A. E. Warner T. A. Guillen L. A. (2021). Accuracy assessment in convolutional neural network-based deep learning remote sensing studies—Part 2: Recommendations and best practices. Remote Sens. 13, 2591. doi: 10.3390/rs13132591 30654563

[B31] Ministry of Trade (2024). Fresh fruit and vegetable sector report (Ankara: Republic of Türkiye Ministry of Trade).

[B32] Nedeljković D. Božić D. Malidža G. Rajković M. Knežević S. Z. Vrbničanin S . (2025). Influence of time of weed removal on maize yield and yield components based on different planting patterns, the application of pre-emergence herbicides and weather conditions. Plants 14, 419. doi: 10.3390/plants14030419 39942981 PMC11820725

[B33] Olsen A. Konovalov D. A. Philippa B. Ridd P. Wood J. C. Johns J. . (2019). DeepWeeds: A multiclass weed species image dataset for deep learning. Sci. Rep. 9, 2058. doi: 10.1038/s41598-018-38343-3 30765729 PMC6375952

[B34] Padilla R. Passos W. L. Dias T. L. B. Netto S. L. Da Silva E. A. B . (2021). A comparative analysis of object detection metrics with a companion open-source toolkit. Electronics 10, 279. doi: 10.3390/electronics10030279 30654563

[B35] Parker C. (2009). Observations on the current status of Orobanche and Striga problems worldwide. Pest. Manage. Sci. 65, 453–459. doi: 10.1002/ps.1713 19206075

[B36] Parven A. Meftaul I. M. Venkateswarlu K. Megharaj M . (2025). Herbicides in modern sustainable agriculture: Environmental fate, ecological implications, and human health concerns. Int. J. Environ. Sci. Technol. 22, 1181–1202. doi: 10.1007/s13762-024-05818-y 30311153

[B37] Prakash V. Srivastva A. K. (2006). Crop-weed competition studies in tomato (Lycopersicon esculentum) under mid-hills of North-West Himalayas. Indian J. Weed. Sci. 38, 86–88.

[B38] Qiu Z. Huang X. Xu X. (2025). Minima-YOLO: A lightweight identification method for lithium mineral components under a microscope based on YOLOv8. Sensors 25, 2048. doi: 10.3390/s25072048 40218561 PMC11991495

[B39] Redmon J. Divvala S. Girshick R. Farhadi A . (2016). You only look once: Unified, real-time object detection. 2016. IEEE Conf. Comput. Vision Pattern Recog. (CVPR)., 779–788. doi: 10.1109/CVPR.2016.91 25079929

[B40] Redmon J. Farhadi A. (2018). YOLOv3: An incremental improvement.

[B41] Regensberg P. L. (2007). A floristic survey of the Baca National Wildlife Refuge, San Luis Valley, Colorado. (Boulder, Colorado, USA: University of Colorado at Boulder).

[B42] Reichstein M. Camps-Valls G. Stevens B. Jung M. Denzler J. Carvalhais N. . (2019). Deep learning and process understanding for data-driven Earth system science. Nature 566, 195–204. doi: 10.1038/s41586-019-0912-1 30760912

[B43] Ren S. He K. Girshick R. Sun J . (2017). Faster R-CNN: Towards real-time object detection with region proposal networks. IEEE Trans. Pattern Anal. Mach. Intell. 39, 1137–1149. doi: 10.1109/TPAMI.2016.2577031 27295650

[B44] Ryu J. Kwak D. Choi S. (2025). YOLOv8 with post-processing for small object detection enhancement. Appl. Sci. 15, 7275. doi: 10.3390/app15137275 30654563

[B45] Shi L. Bai Z. Yin X. Wei Z. You H. Liu S. . (2025). OMB-YOLO-tiny: A lightweight detection model for damaged Pleurotus ostreatus based on enhanced YOLOv8n. Horticulturae 11, 744. doi: 10.3390/horticulturae11070744 30654563

[B46] Skacev H. Micovic A. Gutic B. Dotilic D. Vesic A. Ignjatovic V. . (2020). “ On the development of the automatic weed detection tool”, in: 2020 Zooming Innovation in Consumer Technologies Conference (ZINC) (Novi Sad: IEEE), 123–126. doi: 10.1109/ZINC50678.2020.9161802

[B47] Sudars K. Jasko J. Namatevs I. Ozola L. Badaukis N. (2020). Dataset of annotated food crops and weed images for robotic computer vision control. Data in brief 31, 105833. doi: 10.1016/j.dib.2020.105833 32577458 PMC7305380

[B48] Tao T. Wei X. (2024). STBNA-YOLOv5: An improved YOLOv5 network for weed detection in rapeseed field. Agriculture 15, 22. doi: 10.3390/agriculture15010022 30654563

[B49] Turkish Statistical Institute (TÜİK) (2024). Crop production statistics - vegetables (Tomatoes 2023). (Ankara: Turkish Statistical Institute (TÜİK)).

[B50] Wan G. Ming T. (2025). A lightweight object detection algorithm based on improved YOLOv8. Comput. Artif. Intell. 2, 37–43. doi: 10.70267/cai.25v2n2.3743

[B51] Wang C.-Y. Bochkovskiy A. Liao H.-Y. (2022). YOLOv7: Trainable bag-of-freebies sets new state-of-the-art for real-time object detectors. doi: 10.1109/cvpr52729.2023.00721

[B52] Warwick S. I. Beckie H. J. Thomas A. G. McDonald T . (2000). The biology of Canadian weeds. 8. Sinapis arvensis . L. (updated). Can. J. Plant Sci. 80, 939–961. doi: 10.4141/P99-139

[B53] Zaragoza C. Tei F. Montemurro P. Baumann D. T. Dobrzanski A. Giovinazzo R. . (2003). Weeds and weed management in processing tomato. Acta Hortic. 613, 111–121. doi: 10.17660/ActaHortic.2003.613.13

[B54] Zhao Z.-Q. Zheng P. Xu S.-T. Wu X . (2019). Object detection with deep learning: A review. IEEE Trans. Neural Networks Learn. Syst. 30, 3212–3232. doi: 10.1109/TNNLS.2018.2876865 30703038

[B55] Zhou Q. Li H. Cai Z. Zhong Y. Zhong F. Lin X. . (2025). YOLO-ACE: Enhancing YOLO with augmented contextual efficiency for precision cotton weed detection. Sensors 25, 1635. doi: 10.3390/s25051635 40096500 PMC11902517

[B56] Zhu X. X. Tuia D. Mou L. Xia G.-S. Zhang L. Xu F. . (2017). Deep learning in remote sensing: A comprehensive review and list of resources. IEEE Geosci. Remote Sens. Mag. 5, 8–36. doi: 10.1109/MGRS.2017.2762307 25079929

